# 3-Econsystems: MicroRNAs, Receptors, and Latent Viruses; Some Insights Biology Can Gain from Economic Theory

**DOI:** 10.3389/fmicb.2016.00369

**Published:** 2016-03-23

**Authors:** Hanan Polansky, Adrian Javaherian

**Affiliations:** The Center for the Biology of Chronic DiseaseValley Cottage, NY, USA

**Keywords:** latent virus, microRNA, microcompetition, long non-coding RNA, cis-regulatory element, GA-binding protein

## Abstract

This mini-review describes three biological systems. All three include competing molecules and a limiting molecule that binds the competing molecules. Such systems are extensively researched by economists. In fact, the issue of limited resources is the defining feature of economic systems. Therefore, we call these systems “econsystems.” In an econsystem, the allocation of the limiting molecule between the competing molecules determines the behavior of the system. A cell is an example of an econsystem. Therefore, a change in the allocation of a limiting molecule as a result of, for instance, an abnormal change in the concentration of one of the competing molecules, may result in abnormal cellular behavior, and disease. The first econsystem described in this mini-review includes a long non-coding RNA and a messenger RNA (lncRNA and mRNA). The limiting molecule is a microRNA (miRNA). The lncRNA and mRNA are known as competing endogenous RNAs (ceRNAs). The second econsystem includes two receptors, and the limiting molecule is a ligand. The third econsystem includes a cis-regulatory element of a latent virus and that of a human gene. The limiting molecule is a transcription complex that binds both cis-elements.

This mini-review describes three econsystems: RNAs, receptors, and cis-regulatory elements. All three systems include a limiting molecule that binds the competing molecule. Such systems are extensively researched by economists. In fact, the issue of limited resources is the defining feature of economic systems (Mas-Colell et al., 1995; Mankiw, 2014). This review shows that in all three cases, the allocation of the limiting molecule between the competing molecules determines the behavior of the system. We believe that these systems hold the key to some of the most important questions in biology today.

The first type of econsystem described in this mini-review includes RNAs. Non-protein-coding RNAs, excluding ribosomal and transfer RNAs, were previously thought to be non-functional (Tye et al., 2015). They have been dismissed as background noise, serving no function in the cell. The two major classes of ncRNAs are small ncRNAs (under 200 nt) and long non-coding RNAs (lncRNAs, 200 nt and longer). Furthermore, small ncRNAs are subdivided based on function and cellular location. Subclasses include transfer RNA (tRNA), small nuclear RNA (snRNA), small nucleolar RNA (snoRNA), small interfering RNA (siRNA), piwi-interacting RNA (piRNA), and microRNA (miRNA). LncRNAs are present in a variety of sizes, from just over 200 nt to as long as several kb. MiRNAs regulate the expression of their target genes post-transcriptionally by binding to the messenger RNA (mRNA) (Liz and Esteller, 2016). Consequently, the binding of miRNA to mRNA inhibits translation, or leads to degradation of the target mRNA.

LncRNA also binds miRNA. This binding prevents the miRNA from binding to the mRNA. The lncRNAs and mRNAs that compete for miRNA binding are known as competing endogenous RNAs (ceRNAs). These ceRNAs act as molecular sponges for a miRNA through their miRNA binding sites, referred to as miRNA response elements (MRE). By titrating specific miRNAs, these MREs affect miRNA availability, and, in turn, downstream processes. The binding of a miRNA to MREs within the mRNA leads to translational repression or degradation of the mRNA (Tan et al., 2015). A lncRNA that has higher density of MREs relative to the competing mRNA, and higher concentration, has a significant post-transcriptional effect. Since the concentration of miRNA is limiting, the lncRNA decreases the *availability* of the miRNA to mRNA (see Figure 1A).

**Figure 1:**
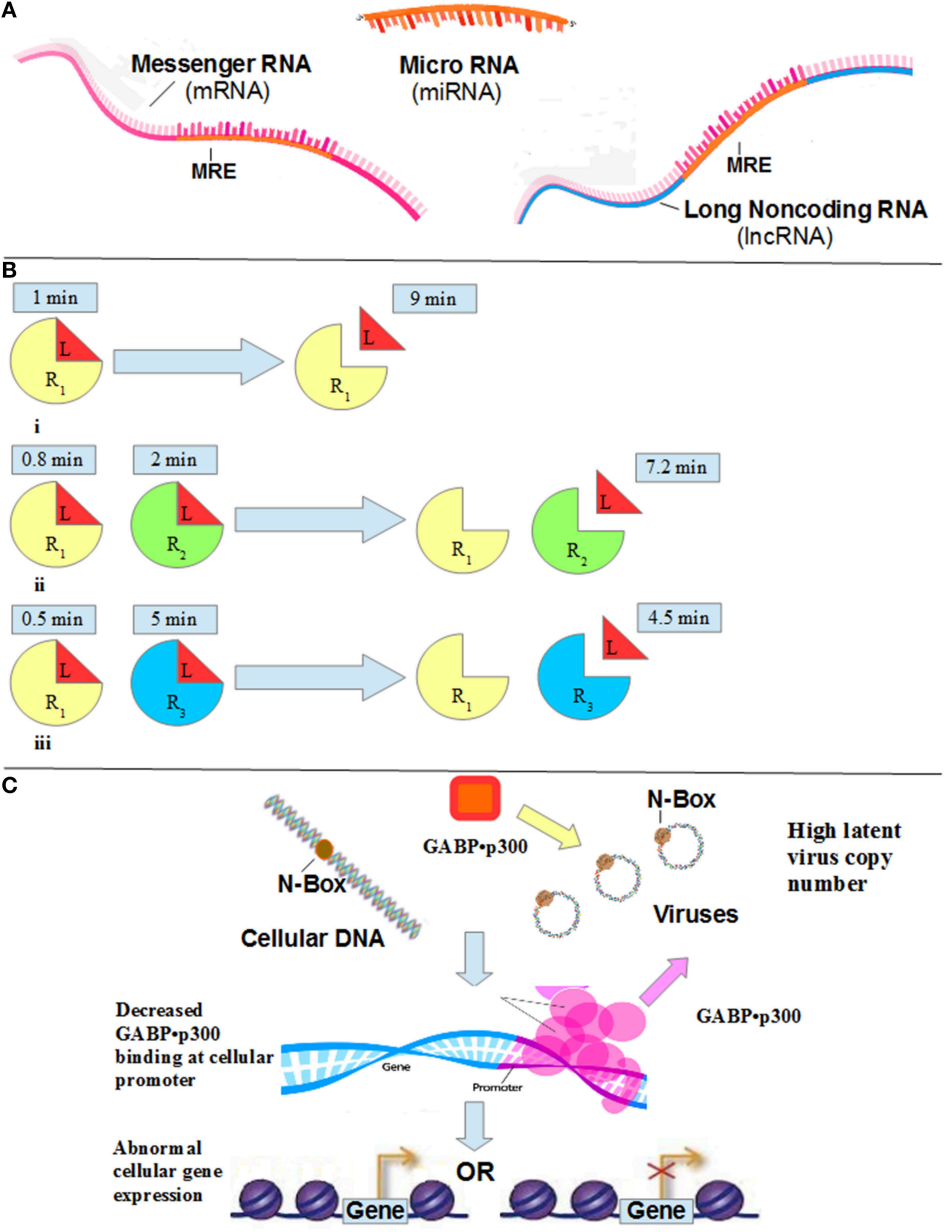
**(A)** Both mRNA and lncRNA have miRNA binding sites (miRNA response elements, or MRE), which bind miRNA. Since the concentration of miRNA is limiting, the lncRNA decreases the *availability* of the miRNA to mRNA. **(B)** Residence Time. When observing the behavior of a ligand, L, for 10 min, (i) in the presence of R_1_ only, the observer sees L bound to the receptor 10% of the time (i.e., 1 min). (ii) When observing the behavior of L in the presence of both R_1_ and R_2_, where R_2_ has 2 times more affinity for the ligand L than does R_1_, the residence time on R_2_ is 2 min, and the residence time on R_1_ is 0.8 min (10% of the time L is free, that is, 8 min). (iii) Consider R_3_ that has a 5 times higher affinity relative to R_1_. When observing the behavior of L in the presence of both R_1_ and R_3_, the residence time of R_3_ is 5 min. The residence time of R_1_ is, therefore, 10% of the time L is free, or unbound, that is, 10% of 5 min, or 0.5 min. **(C)** An increase in latent viral copy number leads to a decrease in GABP•p300 availability to the cellular gene promoter (note the pink arrow). The result is a dysregulation in cellular gene expression.

An example of a ceRNA, and its physiological consequences, involves a lncRNA called colon cancer-associated transcript-1 (CCAT1), which is up-regulated in gallbladder cancer (GBC) tissues (Ma et al., 2015). CCAT1 up-regulates the expression of Bmil, which is the target gene of miRNA-218-5p, by competitively binding the miRNA, promoting the proliferation and invasiveness of GBC cells. CCAT1, therefore, exhibits an oncogenic effect by modulating the availability of miRNA-218-5p, and therefore, the expression of Bmi1. Knockdown of CCAT1 inhibited the proliferation and migration of GBC cells. By “spongeing” miRNA-218-5p, CCAT1 may promote tumor development.

In another study, a ceRNA was identified that regulates cardiac hypertrophy (Wang et al., 2014). In the study, Wang et al. observed that the myeloid differentiation primary response gene 88 (Myd88), which is related to myocardial infarction induced by ischemia/reperfusion, is down-regulated by miR-489. This down-regulation inhibited hypertrophy. The study identified a lncRNA called the Cardiac Hypertrophy Related Factor (CHRF), that acts as a sponge, that is, decreases the availability of miR-489. Consequently, the expression of CHRF induces hypertrophic responses.

In a third study, Cesana et al. identified a muscle-specific lncRNA, called linc-MD1, which has MREs for two specific miRNAs, miR-133 and miR-135 (Cesana et al., 2011). These two miRNAs target two mRNAs encoding for proteins that function in myogenesis: the Myocyte-Specific Enhancer Factor 2C (MEF2C), targeted by miR-135, and the Mastermind-Like-1 (MAML1), targeted by miR-133. Depletion of the linc-MD1 lncRNA decreased the levels of both MAML1 and MEF2C proteins, while over-expression of linc-MD1 increased the protein levels. An increase in miR-133 and miR-135 levels, in conditions of excess linc-MD1, decreased the expression of MAML1 and MEF2C. These observations indicate a direct competition for miRNA binding between linc-MD1 and the mRNAs of MAML1 and MEF2C. The linc-MD1 lncRNA levels are strongly reduced in Duchenne muscle cells, and over-expression in these cells resulted in recovery of both MAML1 and MEF2C synthesis. Linc-MD1, therefore, governs the timing of muscle differentiation and myogenic alterations.

In the three examples above, the miRNA is limiting. Therefore, the concentration of lncRNAs determines the availability of miRNA for binding with its corresponding mRNA. This scarcity of miRNAs turns the system into an econsystem.

The second econsystem described in this review includes competing receptors and a limiting ligand that binds the competing receptors. In this system, the ligand molecules allocate their binding time, or residence time, between the competing receptors according to their affinity.

For example, consider a scenario where “L” is a ligand, “R_1_” is the original receptor to which L binds, and “R_2_” and “R_3_” are competitive receptors that also bind L but with 2 and 5 times higher affinity relative to R_1_, respectively (see Figure 1B). L allocates 10% of its time bound to R_1_. Therefore, if one observes L in the absence of a competitive receptor for 10 min, he discovers L bound to R_1_ for 1 min. In other words, the “residence time” of L on R_1_ is 1 min. If R_2_ is present, L spends 2 min bound to R_2_ (due to a two-fold higher affinity). Therefore, L allocates its residence time differently. Because 2 min is spent bound to R_2_, there is now only 8 min remaining. As mentioned previously, L allocates 10% of its time bound to R_1_. As a result, in the presence of R_2_, L spends only 0.8 min bound to R_1_. Now let us consider a third receptor R_3_. This receptor has a 5 times higher affinity to L compared to R_1_. Therefore, L spends 5 min bound to R_3_, and 0.5 min bound to R_1_. Note that an increase in the affinity of the competing receptor lowers the residence time of the low affinity receptor (see Figure 2A). Since the duration of the signal produced by the L-R_1_ complex is directly related to the residence time of L at R_1_ (Tummino and Copeland, 2008), the signal transduced from L-R_1_ is reduced accordingly.

**Figure 2:**
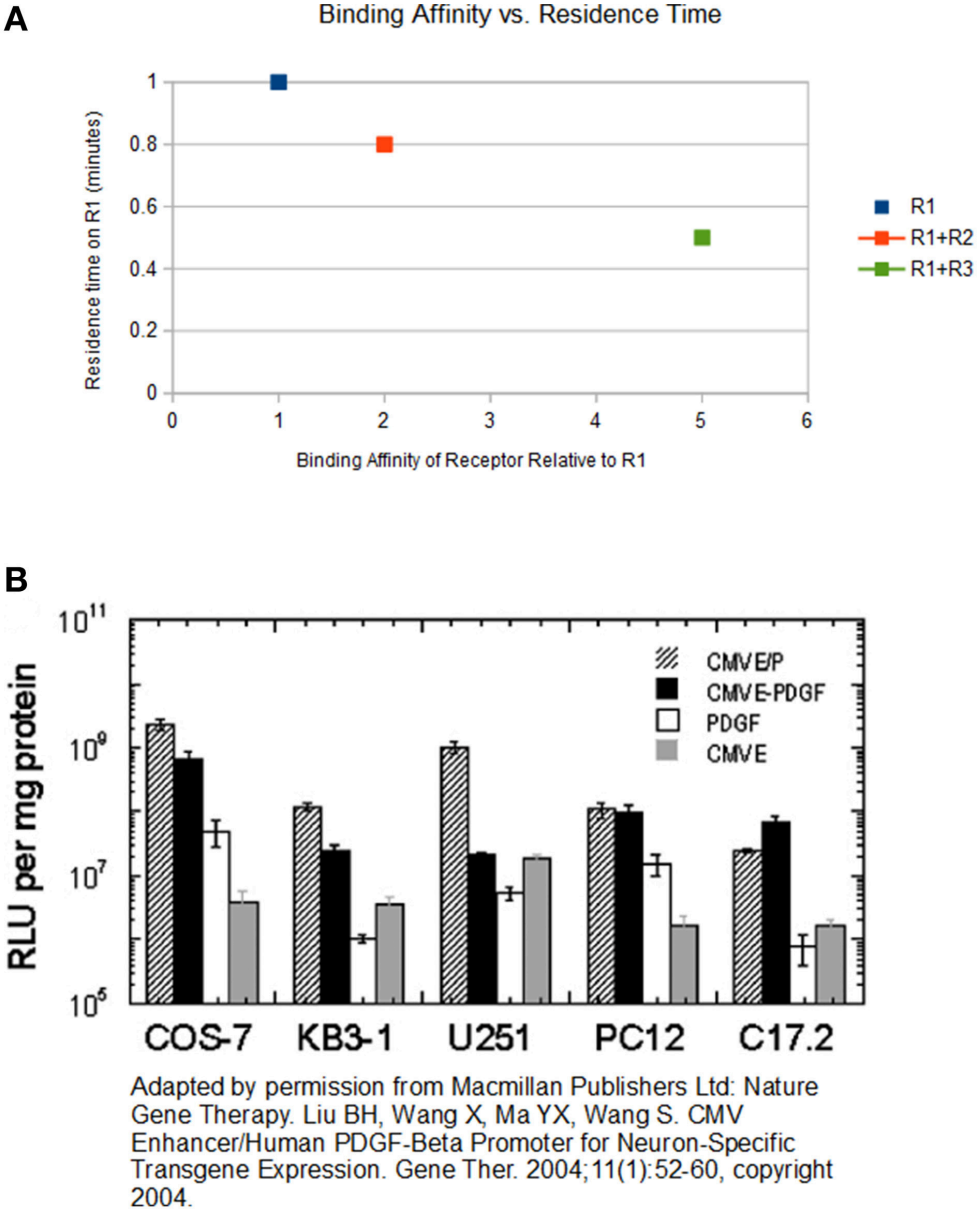
**(A)** The chart above displays the relationship between binding affinity and residence time. The x-axis represents the binding affinity of a receptor, when this affinity is measured without the presence of R_1_. The y-axis represents the residence time of the ligand on R_1_. The first data point represents the residence time of L on R_1_ with no competitive receptors. The second data point represents the residence time of L on R_1_ when both R_2_ and R_1_ are present, and R_2_ has double the affinity of R_1_. The third data point represents the residence time of L on R_1_ when both R_3_ and R_1_ are present, and R_3_ has 5-times the affinity of R_1_. As the binding affinity of the competing receptor increases, the residence time of L on R_1_ decreases. **(B)** Liu et al. reports the expression level driven by the CMV enhancer/promoter (measured in relative light units, or RLU) vs. those driven by the PDGF-b promoter in a variety of cells. Based on the numbers on the y-axis, which are depicted logarithmically, suggests that the CMV P/E is about 150-fold stronger than the PDGF-b promoter.

Note that the negative effect of a higher affinity receptor on the residence time of a lower affinity receptor depends on the existence of displacement asymmetry. To explain this asymmetry, consider a case where two molecules compete for binding to a third molecule. During this competition, the two molecules try to displace each other. Displacement asymmetry says that the probability of displacing the lower affinity molecule by the higher affinity molecule is higher than the probability of displacing the higher affinity molecule by the lower affinity molecule. In other words, higher affinity means more displacing power. The calculations in the example above assume an extreme case where the probabilities are 1 for the higher affinity receptor, and 0 for the lower affinity receptor. The displacement asymmetry has many manifestations. One is binding order. For instance, mineralocorticoid receptors (MR) show a much higher affinity for binding to natural glucocorticoids than glucocorticoid receptors (GR) (Quas and Fivush, 2009). Consequently, in the presence of both MRs and GRs, the MRs bind the glucocorticoids first.

An example of a receptor econsystem involves the chemokine receptors CXCR4 and CXCR7 (Coggins et al., 2014). Both receptors bind the ligand CXCL12. The two receptors are co-expressed under both normal and pathological conditions. Furthermore, both receptors signal through β-arrestin 2 dependent pathways. CXCR7 has a 10-fold higher binding affinity for CXCL12 relative to CXCR4, and therefore CXCR7 functions are enhanced. Coggins et al. (2014) analyzed the dynamics of CXCL12-dependent recruitment of β-arrestin 2 in cells expressing CXCR4 (CXCR4+), CXCR7 (CXCR7+) or both (CXCR4+-CXCR7+). The authors reported that CXCR7 “wins the competition” and that expression of CXCR7 decreased the magnitude and duration of β-arrestin 2 recruitment to CXCR4 and elevated the concentration of CXCL12 required to produce a signal above basal levels. CXCL12 increased recruitment of β-arrestin 2 to CXCR7 and decreased the association of β-arrestin 2 with CXCR4. In other words, CXCR7 sequestered β-arrestin 2 from CXCR4 in cells with both receptors. Since the concentration of ligand is limiting, the receptor with the higher binding affinity decreases the *availability* of the ligand to the receptor with the lower binding affinity.

In another study, Pawig et al. also report that CXCR7 regulates ligand availability for CXCR4 (Pawig et al., 2015). The elevated concentration of CXCL12 required to produce a signal above basal levels, along with the decreased association of β-arrestin 2 with CXCR4, indicate a decreased CXCR4-CXCL12 residence time resulting from the greater binding affinity of CXCR7 (see Figure 2 in the Coggins et al., 2014). Consequently, the signal transduced from CXCR4 decreased as evidenced by the decreased magnitude and duration of β-arrestin 2 recruitment to CXCR4. The CXCL12/CXCR4 axis has been implicated to be involved in hematopoietic stem and progenitor cell (HSPC) homeostasis, progenitor cell survival and proliferation, vascularization during development, and angiogenesis in the context of ischemia. CXCR7 functions as a scavenger receptor for CXCL12, downtuning CXCL12/CXCR4 signaling, and consequently affecting many physiological processes. Wang and Knaut refer to CXCR7 as an efficient chemokine sink due to successful competition with CXCR4 for access to the ligand (Wang and Knaut, 2014).

As described above, *residence time* is an indicator of binding affinity between a ligand and a receptor. However, binding affinity can also be defined by the ligand *spatial distribution*. This is demonstrated in a study by DeWitt et al. (2002). The authors objective was to determine whether differences in epidermal growth factor receptor (EGFR)-binding affinity lead to differences in spatial localization of autocrine epidermal growth factor (EGF) ligands. The authors reported that decreasing the binding affinity of an autocrine receptor/ligand pair decreased ligand capture efficiency when ligand production was limiting for receptor binding. The authors' experimental data demonstrated that cells can use ligand/receptor binding affinity to regulate ligand spatial distribution when autocrine ligand production is limiting for receptor signaling.

Both systems described above include competing molecules and a limiting molecule that binds the competing molecules. In the case of RNAs, the miRNA is limiting. In the case of the receptors, the ligand is limiting. The limiting element transforms the system into an econsystem. In such a system, the allocation of the limiting element between the competing molecules determines the behavior of the system. It is interesting that in the book “Microcompetition with Foreign DNA and the Origin of Chronic Disease,” another econsystem is described. In this econsystem, the competing molecules are cis-regulatory elements, called N-boxes, of latent viruses and cellular genes, and the limiting molecule is the cellular transcription complex GABP•p300 that binds both elements (Polansky, 2003).

The coactivator p300 is a 2414-amino acid protein initially identified as a binding target of the E1A oncoprotein. Cbp is a 2441-amino acid protein initially identified as a transcriptional activator bound to phosphorylated cAMP response element (CREB) binding protein (hence, cbp). p300 and cbp share 91% sequence identity and are functionally equivalent. Both p300 and cbp are members of a family of proteins collectively referred to as p300.

Although p300 and cbp are widely expressed, their cellular availability is limited. Several studies demonstrated inhibited activation of certain transcription factors resulting from competitive binding of p300 to other cellular or viral proteins. For example, competitive binding of p300, or cbp, to the glucocorticoid receptor (GR), or the retinoic acid receptor (RAR), inhibited activation of a promoter dependent on the AP-1 transcription factor (Kamei et al., 1996). Competitive binding of cbp to STAT1α inhibited activation of a promoter dependent on both the AP-1 and ETS transcription factors (Horvai et al., 1997). Competitive binding of p300 to STAT2 inhibited activation of a promoter dependent on the NF-kB Re1A transcription factor (Hottiger et al., 1998). Other studies also demonstrated the limited availability of p300 (Polansky, 2003).

Since p300 is limiting, the GABP•p300 complex is also limiting. Viral promoters/enhancers compete with cellular promoters for this transcription complex. Just like the ceRNA network discussed above, there are two competing entities that compete for the binding of a limiting molecular complex. This competition was called Microcompetition (Polansky, 2003). Consider the following highlights of this genetic econsystem, and the role of latent viruses in this system.

Many viruses consist of an N-box, which is a core binding sequence found in their promoters/enhancers. After establishing a latent infection, the viral N-boxes bind the cellular GABP•p300 transcription complex. Since the complex is limiting, the viral N-boxes decrease the *availability* of the complex to cellular genes (see Figure 1C). As a result, the cellular genes express an abnormal level of their protein. Those that are stimulated by the GABP•p300 complex produce fewer proteins, and those that are suppressed by the complex produce more proteins. The abnormal levels of these cellular proteins can cause a disease.

Latent viruses have been dismissed as harmless, with no effect on the host cellular processes. However, many common viruses, which establish a latent infection, have a strong N-box in their promoters/enhancers. These viruses include the Epstein-Barr virus (EBV), Cytomegalovirus (CMV), Herpes Simplex Virus 1 (HSV-1), Human Immunodeficiency Virus (HIV), and Human T-cell lymphotropic virus (HTLV). It is interesting that the CMV has the strongest promoter/enhancer known to science. To estimate the effect of microcompetition with a latent CMV on the infected cell, one can combine the results from a few studies. Results from Liu et al. (2004) can be used to estimate the strength of the CMV promoter/enhancer, which includes the N-box, relative to the strength of the promoter of the cellular platelet-derived growth factor-b chain (PDGF-b) gene. Liu et al. reports the expression level driven by the CMV E/P (that is, the CMV enhancer/promoter) vs. those driven by the PDGF-b promoter in a variety of cells, including the COS-7, KB3-1, U251, PC12, and C17.2 cells (see Figure 2B). A close inspection of the numbers on the y-axis shown in Figure 2B, which are depicted logarithmically, suggests that the CMV P/E is about 150-fold stronger than the PDGF-b promoter. We suspect that the reason for this difference is the much higher affinity between the CMV promoter/enhancer and the GABP•p300 transcription complex relative to the affinity between the PDGF-b promoter and this complex. Slobedman and Mocarski showed that during latency, an infected cell harbors about 10 copies of the CMV (Slobedman and Mocarski, 1999). Therefore, the impact of a latent infection with the CMV on the *residence time* of the GABP•p300 complex on cellular genes, or the *spatial distribution* of the GABP•p300 complex around cellular genes, is equivalent to the impact created by the introduction of 1500 copies of additional PDGF-b genes into the cell. Adam et al. showed that PDGF-b is susceptible to microcompetition with CMV (Adam et al., 1996). Therefore, according to Microcompetition theory, a latent CMV infection would result in a decrease in PDGF-b transcription followed by a decrease in the concentration of the expressed protein in the latently infected cell, ultimately leading to disease.

In “Microcompetition with Foreign DNA and the Origin of Chronic Disease,” the connection between microcompetition and most major diseases is developed. The tissue factor (TF), CD18, and CD49d genes are all suppressed by GABP. Furthermore, according to the theory, microcompetition between the latent virus and these genes for GABP increases their transcription, thereby increasing the risk of cardiovascular and autoimmune diseases. In contrast, the BRCA1 and retinoblastoma (Rb) tumor suppressor genes are transactivated by GABP and microcompetition between these genes and the latent virus decreases their transcription, increasing the risk of cancer.

We believe that an increase in the number of latent viral promoters/enhancers is the event that triggers the development of most major diseases. In other words, the increase in the number of viral promoters/enhancers is the disruption that shifts the genetic econsystem from normal to abnormal, or from health to disease. There are many events that increase the number of latent viruses in infected individuals. One such event is an increase in the level of stress. In a recent paper, Polansky and Javaherian used the Microcompetition theory to explain how stress can cause breast cancer in women infected with a latent virus (Polansky and Javaherian, 2015). As it turns out, most people harbor a latent viral infection. Seroprevalence of CMV is greater than 70–80% by the age of 50 (Reddehase, 2013). Furthermore, more than 90–95% are infected with the EBV (Green and Michaels, 2013). The HSV-1 has an estimated seroprovalence of greater than 90% in many nations (Bernstein et al., 2013). Therefore, most people should do everything they can to protect their genetic econsystem, and prevent an increase in the number of latent viral promoters/enhancers. Otherwise, according to Microcompetition theory, these people will develop a major disease, such as cancer, heart disease, stroke, diabetes, and many autoimmune diseases.

To summarize, competition for limited resources is a hallmark of economical systems. As we showed in this mini-review, scientists are starting to realize that certain biological systems behave in similar ways. We believe that this discovery is very promising, and has the potential to revolutionize our understanding of the etiology of most major diseases.

## Author contributions

All authors listed, have made substantial, direct and intellectual contribution to the work, and approved it for publication.

### Conflict of interest statement

The authors declare that the research was conducted in the absence of any commercial or financial relationships that could be construed as a potential conflict of interest.

## References

[B1] Adam G. I. Miller S. J. Ulleras E. Franklin G. C. (1996). Cell-type-specific modulation of PDGF-B regulatory elements via viral enhancer competition: a caveat for the use of reference plasmids in transient transfection assays. Gene 178, 25–29. 10.1016/0378-1119(96)00318-6 8921886

[B2] Bernstein D. I. Bellamy A. R. Hook E. W. III Levin M. J. Wald A. Ewell M. G.. (2013). Epidemiology, clinical presentation, and antibody response to primary infection with herpes simplex virus type 1 and type 2 in young women. Clin. Infect. Dis. 56, 344–351. 10.1093/cid/cis891 23087395 PMC3540038

[B3] Cesana M. Cacchiarelli D. Legnini I. Santini T. Sthandier O. Chinappi M.. (2011). A long noncoding RNA controls muscle differentiation by functioning as a competing endogenous RNA. Cell 147, 358–369. 10.1016/j.cell.2011.09.028 22000014 PMC3234495

[B4] Coggins N. L. Trakimas D. Chang S. L. Ehrlich A. Ray P. Luker K. E.. (2014). CXCR7 controls competition for recruitment of β-arrestin 2 in cells expressing both CXCR4 and CXCR7. PLoS ONE 9:e98328. 10.1371/journal.pone.0098328 24896823 PMC4045718

[B5] DeWitt A. Iida T. Lam H. Y. Hill V. Wiley H. S. Lauffenburger D. A. (2002). Affinity regulates spatial range of EGF receptor autocrine ligand binding. Dev. Biol. 250, 305–316. 10.1006/dbio.2002.0807 12376105

[B6] Green M. Michaels M. G. (2013). Epstein-barr virus infection and posttransplant lymphoproliferative disorder. Am. J. Transplant. 13, 41–54. 10.1111/ajt.12004 23347213

[B7] Horvai A. E. Su L. Korzus E. Brard G. Kalafus D. Mullen T. M.. (1997). Nuclear integration of JAK/STAT and Ras/AP-1 signaling by CBP and p300. Proc. Natl. Acad. Sci. U.S.A. 94, 1074–1079. 10.1073/pnas.94.4.1074 9037008 PMC19746

[B8] Hottiger M. O. Felzien L. K. Nabel G. J. (1998). Modulation of cytokine-induced HIV gene expression by competitive binding of transcription factors to the coactivator p300. EMBO J. 17, 3124–3134. 10.1093/emboj/17.11.3124 9606194 PMC1170651

[B9] Kamei Y. Xu L. Heinzel T. Torchia J. Kurokawa R. Gloss B.. (1996). A CBP integrator complex mediates transcriptional activation and AP-1 inhibition by nuclear receptors. Cell 85, 403–414. 10.1016/S0092-8674(00)81118-6 8616895

[B10] Liu B. H. Wang X. Ma Y. X. Wang S. (2004). CMV enhancer/human PDGF-beta promoter for neuron-specific transgene expression. Gene Ther. 11, 52–60. 10.1038/sj.gt.3302126 14681697

[B11] Liz J. Esteller M. (2016). lncRNAs and microRNAs with a role in cancer development. Biochim. Biophys. Acta 1859, 169–176. 10.1016/j.bbagrm.2015.06.015 26149773

[B12] Ma M.-Z. Chu B.-F. Zhang Y. Weng M.-Z. Qin Y.-Y. Gong W.. (2015). Long non-coding RNA CCAT1 promotes gallbladder cancer development via negative modulation of miRNA-218-5p. Cell Death Dis. 6:e1583. 10.1038/cddis.2014.541 25569100 PMC4669740

[B13] Mankiw N. G. (2014). Principles of Economics, 7th Edn. Mason, OH: South-Western College Pub.

[B14] Mas-Colell A. Whinston M. D. Green J. R. (1995). Microeconomic Theory, 1st Edn. New York, NY: Oxford University Press.

[B15] Pawig L. Klasen C. Weber C. Bernhagen J. Noels H. (2015). Diversity and inter-connections in the CXCR4 chemokine receptor/ligand family: molecular perspectives. Front. Immunol. 6:429. 10.3389/fimmu.2015.00429 26347749 PMC4543903

[B16] Polansky H. (2003). Microcompetition with Foreign DNA and the Origin of Chronic Disease. New York, NY: The Center for the Biology of Chronic Disease.

[B17] Polansky H. Javaherian A. (2015). Commentary: the unliganded glucocorticoid receptor positively regulates the tumor suppressor gene BRCA1 through GABP beta. Front. Cell. Infect. Microbiol. 5:66. 10.3389/fcimb.2015.00066 26442220 PMC4585232

[B18] Quas J. A. Fivush R. (2009). Emotion in Memory and Development: Biological, Cognitive, and Social Considerations. New York, NY: Oxford University Press, Inc.

[B19] Reddehase M. J. (2013). Cytomegaloviruses: From Molecular Pathogenesis to Intervention, Vol. 2 Norfolk, UK: Caister Academic Press.

[B20] Slobedman B. Mocarski E. S. (1999). Quantitative analysis of latent human cytomegalovirus. J. Virol. 73, 4806–4812. 10233941 10.1128/jvi.73.6.4806-4812.1999 PMC112523

[B21] Tan J. Y. Sirey T. Honti F. Graham B. Piovesan A. Merkenschlager M.. (2015). Extensive microRNA-mediated crosstalk between lncRNAs and mRNAs in mouse embryonic stem cells. Genome Res. 25, 655–666. 10.1101/gr.181974.114 25792609 PMC4417114

[B22] Tummino P. J. Copeland R. A. (2008). Residence time of receptor-ligand complexes and its effect on biological function. Biochemistry 47, 5481–5492. 10.1021/bi8002023 18412369

[B23] Tye C. E. Gordon J. Martin-Buley L. A. Stein J. L. Lian J. B. Stein G. S. (2015). Could lncRNAs be the missing links in control of mesenchymal stem cell differentiation? J. Cell. Physiol. 230, 526–534. 10.1002/jcp.24834 25258250 PMC4247170

[B24] Wang J, Knaut, H. (2014). Chemokine signaling in development and disease. Development 141, 4199–4205. 10.1242/dev.101071 25371357 PMC4302920

[B25] Wang K. Liu F. Zhou L.-Y. Long B. Yuan S.-M. Want Y.. (2014). The long noncoding RNA CHRF regulates cardiac hypertrophy by targeting miR-489. Circ. Res. 114, 1377–1388. 10.1161/CIRCRESAHA.114.302476 24557880

